# Macphail (1987) Revisited: Pigeons Have Much Cognitive Behavior in Common With Humans

**DOI:** 10.3389/fpsyg.2020.618636

**Published:** 2021-01-21

**Authors:** Thomas R. Zentall

**Affiliations:** Department of Psychology, University of Kentucky, Lexington, KY, United States

**Keywords:** Macphail, comparative cognition, cognitive biases, animal intelligence, pigeons

## Abstract

The hypothesis proposed by Macphail (1987) is that differences in intelligent behavior thought to distinguish different species were likely attributed to differences in the *context* of the tasks being used. Once one corrects for differences in sensory input, motor output, and incentive, it is likely that all vertebrate animals have comparable intellectual abilities. In the present article I suggest a number of tests of this hypothesis with pigeons. In each case, the evidence suggests that either there is evidence for the cognitive behavior, or the pigeons suffer from biases similar to those of humans. Thus, Macphail’s hypothesis offers a challenge to researchers to find the appropriate conditions to bring out in the animal the cognitive ability being tested.

## Introduction

In a classic article, Macphail (1987) made the remarkable claim that differences among vertebrate species in the acquisition of tasks thought to be a measure of intelligence, can be attributed largely to differences in contextual variables. In particular, those contextual differences are likely attributable to differences in the animal’s perception of the task, the motor skills required, or to the animal’s motivation for the rewards involved, rather than to differences in intellect. In comparisons between species, differences in those factors may give the impression of differences in intellectual ability.

An example of how differences in task performance between species can be misleading can be seen readily in research on learning set. Learning set, sometimes referred to as learning-to-learn, is defined as the improvement in discrimination learning that comes with experience with successive discriminations. For example, Harlow (1949) found that when monkeys were given simultaneous discrimination training between pairs of three-dimensional objects, the rate of acquisition improved with as the number of discriminations increased. Specifically, the accuracy of the monkeys on the second trial of a discrimination increased from about 65% correct on early discriminations, to about 98% correct after about 60 discriminations. Thus, after considerable training, based on the outcome of Trial 1, the monkeys appeared to develop a win-stay/lose-shift strategy that they could apply on Trial 2 and thereafter. This strategy has been interpreted as a higher cognitive ability.

When Kay and Oldfield-Box (1965) trained rats on a similar set of discriminations involving three-dimensional objects, the rats improved to only about 75% correct on Trials 2–10 after 78 discrimination problems. Based on this difference in findings, and consistent with one’s intuitive belief about the natural order of animal intelligence, one might conclude that monkeys are more intelligent than rats.

Not long after, however, Slotnick and Katz (1974) reasoned that the visual system of the rat may not be ideal for learning visual discriminations. They reasoned that rats might do better with such learning if the discriminations were better suited to their sensory abilities. They tested this hypothesis by giving the rats a series of olfactory discriminations and found that the rate at which the rats learned the successive discriminations improved much faster than with visual discriminations and rivaled that of the monkeys.

The task for psychologists who study comparative cognition is to find the methodology best suited for the species studied. In a sense, one needs to find the best input, output, and motivational conditions appropriate to the animal. The problem, of course, is how to know when one has found the ideal set of variables for the species being studied. How can experimenters take the perspective of the animal? How does one decide that the species does not have a particular capacity?

One approach is to view Macphail’s hypothesis as a challenge. Macphail’s hypothesis can serve as a useful model for an approach to the study of comparative cognition. A good rule of thumb is, when designing an experiment to test for an animal’s cognitive capacity, one should attempt to consider the task from the stimulus, response, and motivational perspective of the animal.

Most of my research has been done with pigeons. I have chosen pigeons, in part, because they are highly visual animals and it is relatively easy to manipulate colors and shapes that are quite easy for them to discriminate. Also, pigeons naturally peck for their food, so pecking at the stimuli is relatively easy for them to learn. Finally, as they are granivors it is relatively easy to motivate them with grain as a reinforcer.

In the remainder of this paper I will describe several of the presumed cognitive abilities attributable to humans (and sometimes to non-human primates) and describe how we have attempted to ask if pigeons too have at least some of this ability. The set of abilities described in this article is not meant to be comprehensive. It is merely a sample of the cognitive abilities that I have studied. Furthermore, it is not meant to examine the comparable ability of other species. The purpose of this enumeration of cognitive abilities is merely to show some of the breadth of competencies that can be found in one particular species, the pigeon. Most of this research was conducted in an operant box with stimuli projected on pecking keys and reinforcement provided from a mixed grain feeder. The conclusion that I have come to in conducting these lines of research is that Macphail’s hypothesis has a lot to be said for it. Furthermore, I am pleased to admit that pursuing this approach to comparative cognition research has been a very rewarding experience.

## Comparative Cognition

### The Sameness Concept

The typical method to assess concept learning in animals is to train them with one set of stimuli and ask if they can apply that conceptual rule they have learned to new stimuli. For example, pigeons can easily learn a task called matching-to-sample with colored stimuli, a task that has the potential to develop a sameness rule. This research generally involves an operant box with three pecking keys. The stimulus is projected on middle key is the sample and the stimuli projected on the two side keys are the comparison stimuli. Specifically, for example, if the sample is red, choice of the red comparison stimulus is reinforced, if the sample is green choice of the green comparison stimulus is reinforced. To test for a sameness rule, one should transfer the pigeons to novel stimuli. We have found that when pigeons are transferred to novel blue and yellow stimuli, there is some evidence of positive transfer (Zentall and Hogan, 1974). However, it is possible that stimulus generalization between the training colors and the testing colors played a role in the transfer found. More convincing evidence was found when the training was with shapes and the transfer task involved colors (Zentall and Hogan, 1976). But there is an inherent problem with transfer designs that involve novel stimuli. Pigeons tend to be neophobic and there is generally a substantial initial disruption of matching accuracy that could be attributed to the novelty of the transfer stimuli.

**FIGURE 1:**
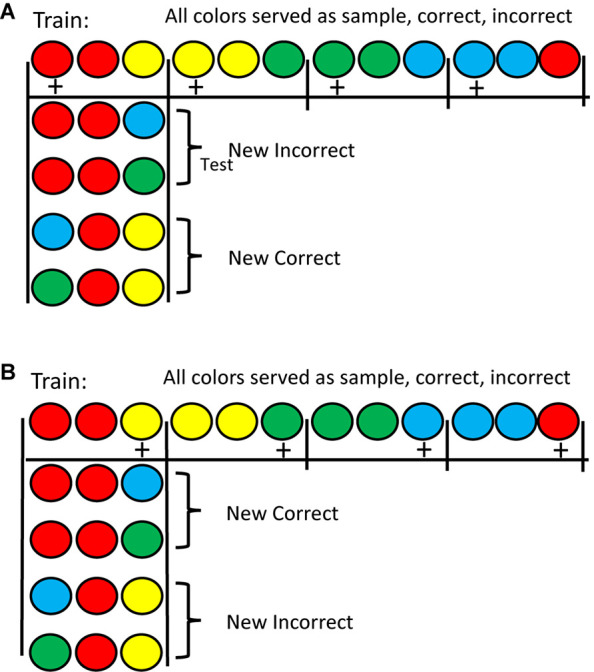
**(A)** Half of the pigeons were trained on matching with the stimuli on the top line (counterbalanced for position correct). Pigeons pecked the center of the three stimuli 10 times to produce the two comparison stimuli. A single peck to either comparision stimulus terminated the trial. Reinforcement is indicated by a +. Testing was done with New Incorrect stimuli and with New Correct stimuli (as shown). The figure shows the red sample test trials. There were also similar test trials with the other three colors (not shown). **(B)** The remaining pigeons were trained on mismatching with the stimuli on the top line (counterbalanced for position correct). Testing was done with New Incorrect stimuli and with New Correct stimuli (as shown). The figure shows the red sample test trials. There were also similar test trials with the other three colors (not shown). After Zentall et al. (2018).

**FIGURE 2:**
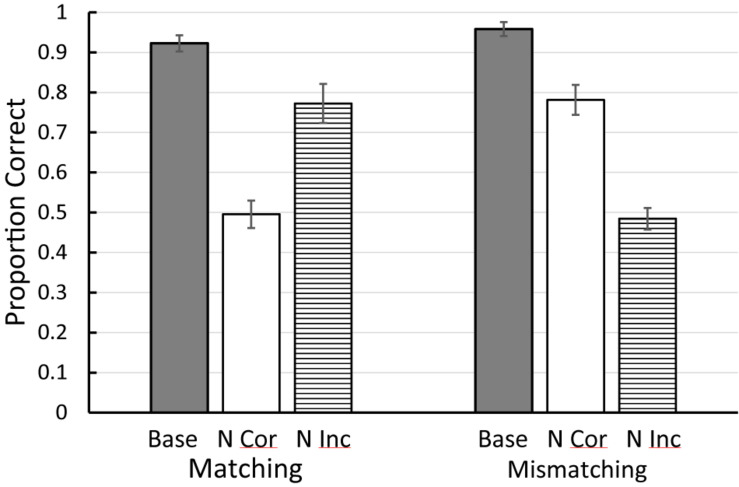
Results of the Zentall et al. (2018) experiment (see Figures 1, 2 for the design). Base = baseline matching and mismatching accuracy. N Cor = accuracy on new correct test trials. N Inc = accuracy on new incorrect test trials. Error bars = ± 1 standard error of the mean.

An alternative approach was attempted by Zentall et al. (2018). They trained pigeons on either a matching or mismatching task with four colors. In training, although each color served as a sample and as the matching comparison, with each sample only one color served as the mismatching stimulus (see Figure 1 for the design of this experiment). This meant that all four colors had served as sample, correct, and incorrect stimulus in one of the four matching problems. Following training, on test trials, either the matching or the mismatching comparison color was replaced with a familiar color but one that was never before seen with that sample. Results for the matching task were as one might expect. Replacing the matching stimulus resulted in a sharp drop in accuracy, whereas replacing the mismatching stimulus resulted in only a small drop in accuracy. The results with the mismatching task, however, were surprising (see Figure 2). Replacing the mismatching stimulus (the correct stimulus from training) resulted in only a small drop in matching accuracy, whereas replacing the matching stimulus (the incorrect stimulus from training) resulted in a large drop in matching accuracy. These results were not only unexpected but are inconsistent with Skinner’s (1950) prediction that all conditional discriminations (including matching and mismatching) involve the learning of simple sample-correct-comparison stimulus-response chains. The results of Zentall et al. (2018; see also Zentall et al., 1981) suggest that the pigeons use the matching stimulus as the basis of choice in both the matching and mismatching tasks. In matching, they locate the matching stimulus and choose it. In mismatching, they locate the matching stimulus and avoid it. Thus, the matching relation between stimuli determines how pigeons learn both of these conditional discriminations and thus, the sameness relation is important for the pigeon.

### Prospective Coding

In Pavlovian conditioning animals are able to anticipate the arrival of biologically important events (e.g., food or shock). Humans, however, have the ability to anticipate the arrival of events and use those anticipations as the basis for making decisions. Humans have the ability to imagine the outcome that they expect to experience. What about other animals?

Trapold (1970) found that in a conditional discrimination, if each sample-correct-comparison chain is followed by a distinctive outcome (e.g., food or water) the anticipation of that outcome can serve as a stimulus to facilitate comparison choice. This phenomenon is known as the differential outcomes effect. Differential outcomes have also been found to improve memory in a delayed matching task. For example, if a delay is inserted between the offset of the sample and the onset of the comparison stimuli, pigeons appear to be able to use the expected outcome as the basis for comparison choice, even when the sample itself is forgotten (Peterson, 1984).

**FIGURE 3:**
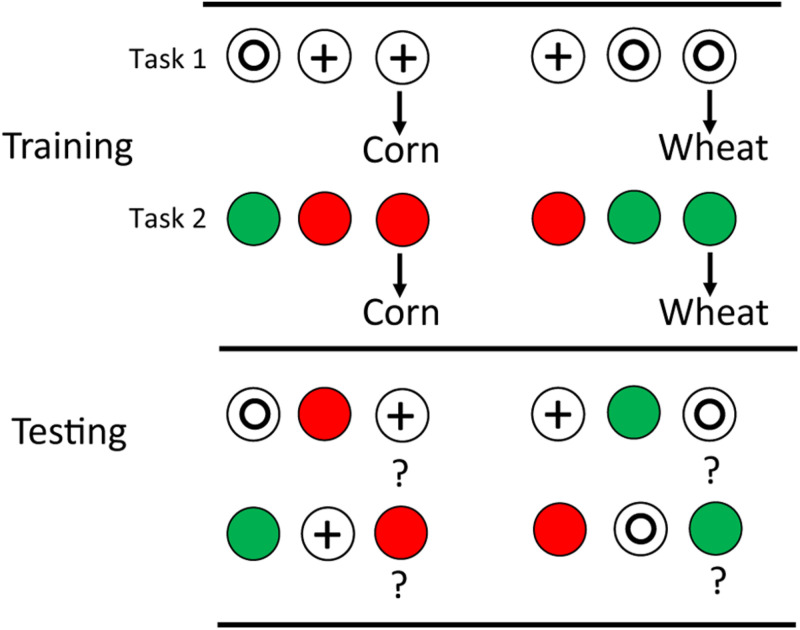
Pigeons were trained on two matching tasks: red green and circle plus with differential outcomes (corn for one trial type, wheat for the other). They were then tested with the samples from one task and the comparisons from the other task. Significant transfer indicated that outcome anticipation based on the samples could serve as discriminative stimuli for comparison choice (after Edwards et al., 1982).

Even more impressive, one can train pigeons on two matching tasks with similar differential outcomes on each (e.g., in each discrimination corn follows correct choice of one comparison, while wheat follows correct choice of the other; see design in Figure 3). If on transfer tests, the sample stimuli are exchanged between the two tasks, it can be shown that outcome associations provide the sole basis for choice of the comparison stimulus (see e.g., Edwards et al., 1982).

Further evidence for anticipatory memory comes from research with the radial maze. In the radial maze, the animal is placed on a central platform and there is food in each of 8, 12, or more arms of the maze. Rats should be motivated to enter each arm once to eat the food there and not repeat arm entries and they generally do so. In fact, to produce errors one must insert a delay at some point in the trial. But how do they keep track of the arms with few repeat entries (errors) as they proceed through the trial? There is evidence that rats start by remembering the arms already entered, but once they have entered half of the arms, they switch to anticipate the arms not yet taken (Cook et al., 1985). If the rats were remembering only the arms already taken, one would expect the probability of an error to increase as a function of the number of arms already taken because of the increasing memory load. Although the probability of an error does initially increase as more arms are visited, it then decreases as the number of arms not yet visited decreases. These data demonstrate that the rats use an efficient strategy for visiting the arms by minimizing the memory load as they proceed through the trial.

Interestingly, pigeons show a similar effect in an operant analog of the radial maze involving pecking keys on a panel (Zentall et al., 1990). In this task, the pigeons must peck each key for reinforcement but on any trial, reinforcement is not provided if the pigeon returns to an already pecked key. Once again, if a delay is inserted either early in the trial or late in the trial the error rate is quite low, but if the delay is inserted toward the middle of the trial the error rate is considerably greater.

### Acquired Equivalence

In an operant box, pigeons can learn a conditional discrimination in which there is an arbitrary relation between the sample and the correct comparison stimulus. When two sample stimuli (e.g., a red light and a vertical line) are each associated with a common comparison stimulus (e.g., a circle), there is a many-to-one mapping of samples on to the same comparison stimulus. Under these conditions one can ask if an equivalence relation develops between the two samples. That is, do those two samples come to *mean* the same thing.

**FIGURE 4:**
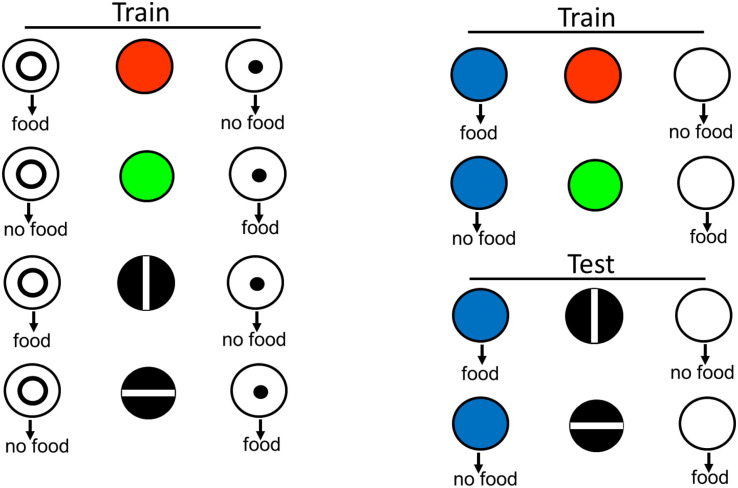
Pigeons were originally trained to choose the circle when the sample was red or a vertical line, and to choose the dot when the sample was green or a horizontal line. To determine if red and vertical were commonly coded and green and horizontal were commonly coded, the pigeons were then trained to choose blue when the sample was red and to choose white when the sample was green. They were then tested with vertical and horizontal line samples and blue and white comparison stimuli. Evidence for functional stimulus equivalence was choice of blue when the sample was a vertical line and white when the sample was a horizontal line (after Urcuioli et al., 1989).

There are several ways to test for equivalence. In one design, the red light is now associated with a new comparison stimulus (e.g., a blue light). The test for equivalence is to ask if, without further training, the vertical line is also associated with the blue light, in spite of the fact that the vertical line and the blue light had never been presented together before (see design in Figure 4). Using this design, Urcuioli et al. (1989) found that pigeons showed positive transfer to those stimuli never presented together before. This finding suggests that for the pigeon, the red light and the vertical line have come to be similarly represented.

Later research attempted to determine the nature of the common representation by inserting a variable duration delay between the offset of the sample and the onset of the comparison stimuli (Friedrich et al., 2004). This research took advantage of the fact that earlier research had found that colored samples were remembered better than line orientations. That is, the forgetting function for line orientation samples was steeper than for colored samples. When a red light and a vertical line were both associated with the same comparison stimulus, however, the slopes of the resulting retention functions suggested that the two samples were commonly represented during the delay. Furthermore, other research suggested that the representation was likely the sample that was easiest to remember (Zentall et al., 1995). So presumably, the pigeons represented the vertical line sample as a red sample, a stimulus that earlier research had indicated was easier to remember.

### Directed Forgetting

When humans are shown a list of words and are told that they will have to remember some of them but not others, they don’t remember as well the words they were told they could forget, as the words they were told they would have to remember (see e.g., Golding and MacLeod, 1998). The implication of this finding is that there is an active rehearsal process triggered by the instruction to remember and the rehearsal process is not triggered by the instruction to forget.

It is often assumed that animals do not have active control over their memory. It is thought that events are remembered and forgotten automatically as a function of the passage of time or intervening events. The challenge in assessing directed forgetting in animals is how to give them *instructions* to remember or forget.

The first presumed evidence of directed forgetting in pigeons was reported by Maki (1981), who used a delayed matching task. Once pigeons had learned to match with delays, on some trials, a stimulus was presented during the delay and on those trials, the comparison stimuli were omitted. Thus, one can think of the delay stimulus as a cue to forget because on those trials, there would not be a test of memory for the sample. As with humans, the test of directed forgetting occurred when, on infrequent probe trials with the forget cue, comparison stimuli *were* presented. In several experiments, pigeons performed very poorly on those probe trials, suggesting that their memory was impaired. Thus, the results suggested that memory for the sample was not automatic.

An important problem with that design, however, is that the forget cue signaled not only the absence of a comparison stimulus test, but also the absence of the possibility of reinforcement on that trial. Thus, because of its association with the absence of reinforcement, the forget cue likely became an aversive stimulus, with all of the accompanying inhibitory affects associated with such a stimulus.

There are several ways to avoid that problem. For example, Roper and Zentall (1994) trained pigeons on a delayed matching task with red and green stimuli and when inserting a forget cue in the delay, followed the forget cue with a simultaneous discrimination involving stimuli different from the matching task (e.g., vertical and horizontal line orientation stimuli in which the vertical lines were always correct). Thus, the forget cue still signaled that the sample could be forgotten but it also indicated that reinforcement (in the form of the simple simultaneous discrimination) would follow. Then, on probe trials, once again, the forget cue was presented followed by the comparison stimuli from the matching task. Results indicated, however, that with this procedure there was little evidence of directed forgetting. That is, the pigeons matched with no loss of accuracy on the probe trials.

**FIGURE 5:**
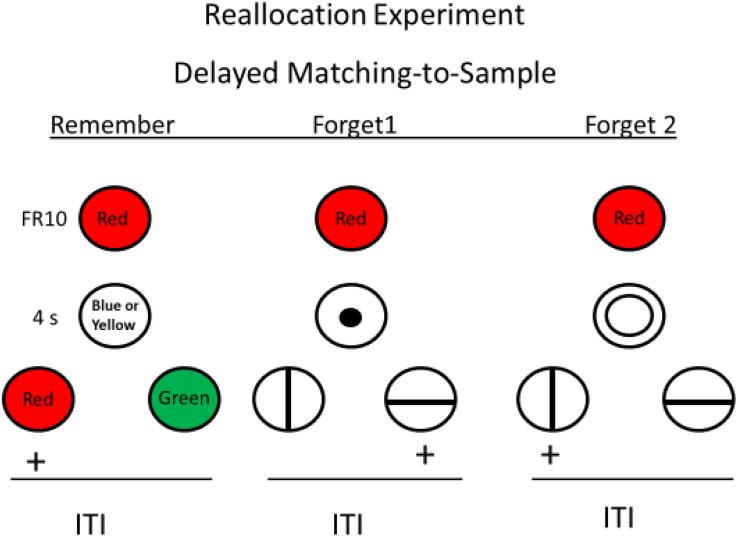
Directed forgetting training with pigeons on red sample trials (green sample trials are not shown). Blue or yellow stimulus presented during the delay signaled sample memory would be tested (remember cue). Vertical or horizontal line presented during the delay signaled signaled sample memory would not be tested (forget cue) but memory for the fortget cue would be tested. Probe trials involved a sample, followed by a forget cue, followed by test (red and green comparison stimuli). Pigeons were significantly less accurate on probe trials than on remember trials (after Roper et al., 1995).

Roper et al. (1995) reasoned that perhaps when the forget cue signaled that a simple simultaneous discrimination would follow, the *instruction* to forget the sample may have been ineffective because the memory load was insufficient to produce forgetting. In human directed forgetting research, being told to forget a word allows the subject time to rehearse other words that they were told to remember. Using this idea, Roper et al. created an analogous task for pigeons in which the forget cue(s) actually served as the sample for another matching task (see Figure 5). With this procedure, the presence of the forget cue instructed the pigeon to forget the sample but remember the forget cue because memory for the forget cue would be required for reinforcement. Thus, the appearance of the forget cue should cause the pigeon to *reallocate* its memory from the sample to the forget cue itself. On probe trials, in which the forget cue was followed by the comparisons appropriate to the sample, the pigeons showed significant forgetting of the sample. Thus, the pigeons showed significant directed forgetting, evidence that under appropriate conditions, they have at least some direct control over what they remember.

### Factors Affecting Oddity Learning

In a mismatching task, reinforcement is provided for choice of the comparison stimulus that does not match the sample. A related task is oddity, in which three stimuli are presented and reinforcement is provided for choice of the stimulus that does not match the other two. The two tasks differ in important ways. In mismatching the sample always appears on the center key, and the pigeons must peck the sample several times before the two comparison stimuli are presented. In oddity, there is no sample (thus no sample pecking) and the odd stimulus can appear on any of the three response keys.

Zentall et al. (1974) compared the pigeon’s acquisition of a two-color mismatching task, with and without required responding to the sample, and oddity in which the odd stimulus could appear on the center key. They found that mismatching was acquired quickly with sample responding required but only slowly without responding to the sample. In the same experiment, they also found little learning of the oddity task. Correct responding by chance on the three-key oddity task is 33% correct and the pigeons generally improved to 50% correct by developing a color preference. Yet, after that, they showed little evidence of learning to select the odd stimulus.

Zentall et al. (1980a) asked if increasing the number of matching stimuli from two to four would affect pigeons’ acquisition of the oddity task. Although increasing the number of matching stimuli decreased the probability being correct by chance to 20%, surprisingly, they found that when the odd stimulus was part of a five-stimulus array, the pigeons acquired the task rapidly. It appears that with four matching stimuli, the odd stimulus stood out better from the “background” of matching stimuli.

Zentall et al. (1980b) tested this hypothesis further, using an array of 25 stimuli, with 24 matching stimuli and one odd stimulus. Although the probability of choosing the correct location by chance was now only 4% and of choosing the correct color by chance was only 50%, the pigeons learned this task very quickly. When such a phenomenon has been reported in humans it has been referred to as *visual pop out* (Treisman, 1985). Although this phenomenon might be considered perceptual rather than cognitive, it is another example of a similarity between humans and other animals. Simple learning theory would predict that with 25 possible response locations, the oddity task would be harder than with only three locations—certainly it should not be any easier.

### Timing

In our modern culture, time plays a very important role. Our ability to keep track of the passage of time, however, is not very good. To aid us, we use watches, clocks, and smart phones. When we were hunter gatherers and until quite recently, external cues like where the sun was in the sky, day/night cycles, and the phases moon, were sufficient because short time intervals were likely not very important. What about other animals? To what extent are they able to discriminate the passage of time?

One measure of short-interval animal timing is the performance of an animal on a fixed interval schedule. For example, if a pigeon receives a reinforcer for the first response after 1 min, with adequate training, one typically sees what has been called a fixed interval *scallop*. Responding does not start immediately after the last reinforcer but then increases, first slowly and then faster, as the time since the last reinforcer approaches 1 min. To get a better measure of the animal’s timing ability one can start the fixed interval trial with the onset of a stimulus and turn it off with reinforcement. After some training, one can introduce *empty* trials in which reinforcement is omitted but the stimulus stays on. If one averages the pecking over a series of such empty trials, the plot of response rate as a function of time since the start of the trial has a peak very close to the time that the reinforcer would have occurred on a fixed interval trial.

Another measure of short-interval animal timing involves the use a temporal discrimination. For example, for pigeons, after having experienced a short interval sample (e.g., 2 s), choice of the red comparison would be reinforced, whereas having experienced a longer sample (e.g., 8 s) choice of the green comparison would be reinforced. After sufficient training, to get an idea of the underlying scale of time for the pigeon, one can present the pigeon with sample durations between 2 and 8 s. The psychophysical function that results when plotting the probability of a *long* response, as a function of the sample duration, provides a measure of the animal’s scale of timing. In particular, the sample duration to which the animal distributes its responses equally between *short* and *long* is referred to as the *point of subjective equality*. Although one might expect that point to be the arithmetic mean of the two training durations (in this case 5 s), it is typically closer to the geometric mean (4 s), suggesting that the pigeons’ judgment of the passage of time is not linear but is logarithmic. In the example given, the geometric mean is at 4 s because the ratio of 2–4 is the same as the ratio of 4–8. Similar psychophysical function have been found for humans.

#### Do Animals Represent Time Categorically?

**FIGURE 6:**
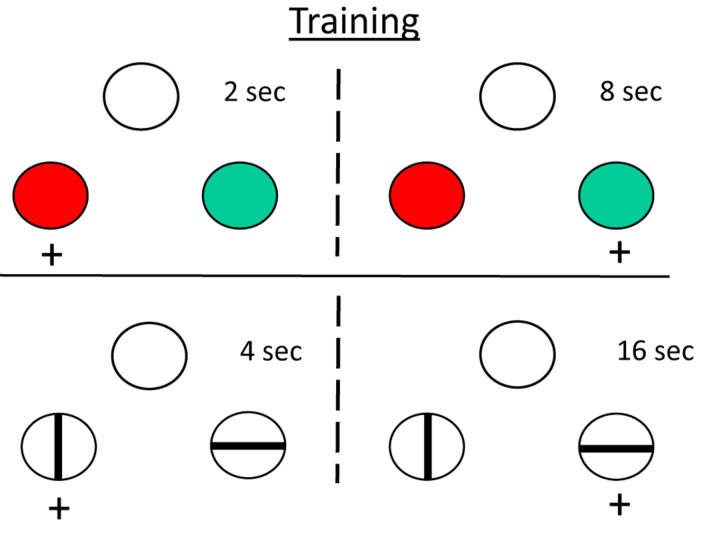
Relative timing experiment (Zentall et al., 2004). Pigeons were trained to discriminate 2 s samples from 8 s sample (top) and 4 s samples from 16 s samples (bottom). They were then tested with 4 and 8 s samples and the comparison stimuli from the other discrimination. Although those sample durations were at the geometric mean of the other discrimination, the pigeons tended to choose “short” (red) when the sample was 4 s and choose “long” (horizontal) when the sample was 8 s.

When humans are given a temporal discrimination like the one described for pigeons above, they are very likely to describe the intervals relationally, as *short* and *long*, rather than in terms of their absolute duration (2 and 8 s). We were interested in whether pigeons also represent intervals relationally as *short* and *long* (Zentall et al., 2004). To answer this question, we trained pigeons on two temporal discriminations, one involving 2 and 8 s samples (with red and green comparison stimuli), and the other involving 4 and 16 s samples (with vertical and horizontal stripes). Note that the 4 s sample falls at the geometric mean of the 2–8 s discrimination, and the 8 s sample falls at the geometric mean of the 4–16 s discrimination (see Figure 6). On probe trials, we presented the 4 s sample with the comparisons from the 2 to 8 s discrimination and the 8 s sample with the comparisons from the 4 to 16 s discrimination.

Normally, presenting durations that correspond to the geometric mean should result equal choice of *long* and *short*. If the pigeons represented the 4 s sample as *short*, however, they might be expected to choose the colored comparison associated with the short, 2 s sample. And if the pigeons represented the 8 s sample as *long*, they might be expected to choose the line comparison associated with the long, 16 s sample. In fact, such a bias was found. Thus, similar to humans, pigeons show some evidence of representing time intervals relationally.

#### Is Subjective Time Affected by What the Animal Is Doing?

As noted earlier, we humans are not very good at estimating the passage of time. For example. when taking an exam, students are often surprised at how much time has elapsed since the start of the exam (time flies when one is cognitively involved). On the other hand, if students are attending a boring lecture, time seems to pass very slowly. Do animals experience a similar effect? Does time pass by faster when pigeons are behaviorally involved than when they are not?

**FIGURE 7:**
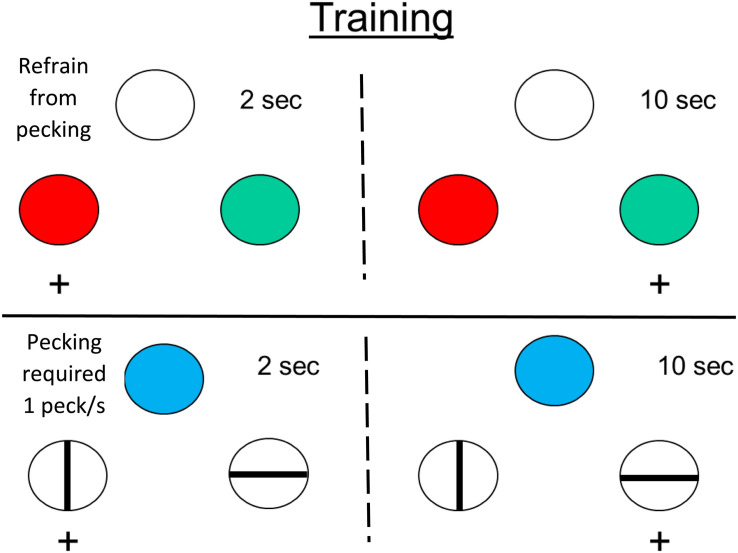
Pigeons were trained on two temporal discriminations involving 2 and 10 s samples. When the sample was white, the pigeons were required to refrain from pecking it. When the sample was blue, the pigeons were required to peck it (once per s). On test trials, when durations between 2 and 10 s were presented. The pigeons tended to treat the white sample durations as longer than the blue sample durations (after Zentall and Singer, 2008).

**FIGURE 8:**
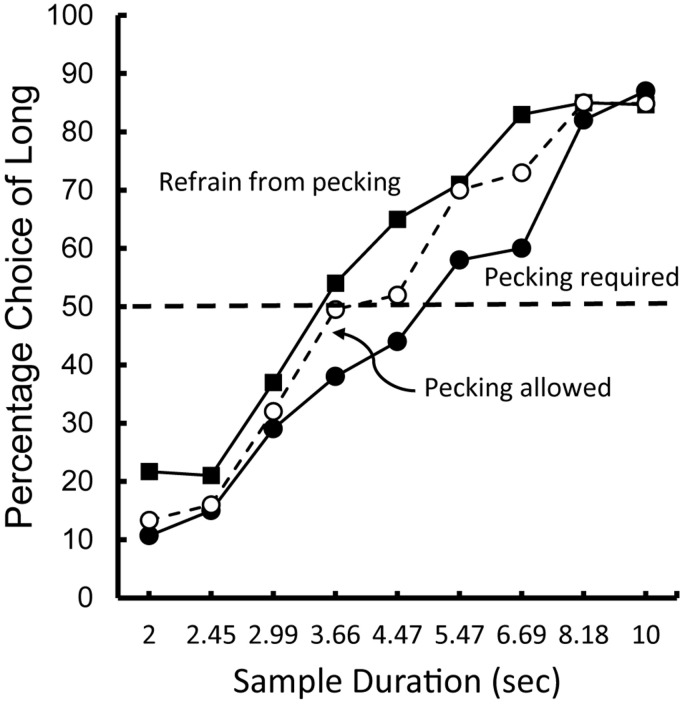
Choice of “long” as a function of sample duration. Pigeons were trained to discriminate between samples of 2 and 10 s. When the sample was white the pigeons were required to refrain from pecking. When the sample was blue the pigeons were required to peck at a rate of 1 peck per s. On test trials, the sample duration was varied between 2 and 10 s. For comparison purposes data are also presented from pigeons for which pecking was allowed. After Zentall and Singer (2008).

To test this possibility, Zentall and Singer (2008) trained pigeons on a temporal discrimination involving 2 and 10 s samples. When the samples were white, the pigeons were required to refrain from pecking, but when the samples were blue, the pigeons were required to peck them at least once per sec (see Figure 7). On test trials, white and blue samples were presented for durations between 2 and 10 s. The question of interest was the effect that sample pecking (and the absence of pecking) had on the psychophysical function (relating choice of long to sample duration), and specifically on the point of subjective equality (see Figure 8). Relative to a group of pigeons that were free to peck or not, they found that when the pigeons were required to peck the temporal samples, the point of subjective equality shifted to longer durations. That is, the pigeons judged that less time had elapsed. Whereas, when the pigeons were required to refrain from pecking the temporal samples, the point of subjective equality shifted to shorter durations. That is, the pigeons judged that more time had elapsed. These results indicate that animals judge the passage of time with biases similar to those of humans. The implication of this research is that, much like humans, pigeons appear to judge the passage of time in terms of the rate at which relevant events occur.

### Counting

The ability to count or to use the number of objects or events as a cue is a quality that adult humans perform routinely and efficiently. However, the degree to which non-verbal organisms have this ability is more controversial. Although relative numerosity judgments have been studied extensively in animals (see e.g., Meck and Church, 1983), absolute numerosity judgments are quite a bit more difficult. After extensive training, Xia et al. (2000) had some success in training pigeons to respond a fixed number of times, defined by the specific Arabic numeral displayed.

In keeping with MacPhail’s suggestion that it is important to find the context appropriate to the animal, Seligman and Meyer (1970) found that after rats had been trained to press a lever for occasional delivery of food, in each session they were delivered exactly three shocks, randomly spaced throughout the session. The introduction of shocks produced suppression in responding; however, once the rats had had some experience with this procedure, they began responding at a higher rate after the third (last) shock had been administered. Thus, they understood when shocks would no longer occur. Similarly, Capaldi and Miller (1988) found that rats trained on a series of four straight-alley runs, in which a reinforcer was found on the first three runs but not on the fourth, ran slower on the fourth run. In both cases rather than asking the animal to count the number of responses they made, these studies had the animals count the number of biologically meaningful events (food). Furthermore, rather than use a discrete measure of counting, they used a continuous measure, response rate or running speed.

We took a similar approach and asked if pigeons could learn that they would be fed after each of the first three 10-peck sequences in a trial, but not after the fourth (Rayburn-Reeves et al., 2010). We used the time to complete each 10-peck sequence as a measure of their counting ability and found that the pigeons completed the 10-peck requirement relatively quickly for each of the first three sequences (about 5.5 s per sequence) but they took almost twice as long to complete the fourth sequence. When one is assessing an animal’s ability to keep track of successively experienced events it is important to control for the time it takes to experience the events because the animals may be judging the passage of time instead of the number of events that it experienced. To control for time between the start of a trial and the fourth sequence (timing rather than counting) we started random trials with a non-reinforced 10-peck sequence. Thus, on those trials, the third reinforced sequence actually occurred at a time when non-reinforcement would have occurred on a standard four-sequence trial. The large increase in latency to complete fifth sequence suggested that the pigeons were counting the number of reinforcers rather than the time from the start of the trial. This line of research demonstrates that tapping into the appropriate motivational system may be critical in assessing the cognitive ability of an animal.

### Imitation

It is well known that some animal are behavioral copiers. We even acknowledge copying by animals with expressions such as to “ape” someone and “monkey see monkey do.” But what psychological processes are involved in the copying of behavior? Piaget (1962) suggested that true imitation involves being able to take the perspective of another. That is, something like “if I put myself in his place, what would I have to do to get the outcome that he is getting.” It is difficult to imagine a young child who is imitating an adult reasoning of that kind and certainly not a non-verbal animal. But the question of whether animals are capable of imitating the behavior of a conspecific was of interest to us.

One could start by asking if an animal could learn a response after seeing another animal perform that response. But of course, one should ask, relative to what. Relative to an animal learning on its own by trial and error, perhaps. But the mere presence of another animal might facilitate learning (an effect known as social facilitation). Furthermore, if the imitation involved the manipulation of an object, the sight of that manipulation could attract the observer to that object (a phenomenon known as stimulus enhancement). Finally, facilitated acquisition could be attributed to what developmental psychologists call learned affordances, learning how the environment works, independent of the action that led to the result (e.g., learning that the up and down movement of a lever leads to the appearance of food). The question is how to test for *true imitation* while controlling for these other presumably less cognitive mechanisms.

Zentall et al. (1996) used a method they referred to as the two-action procedure to control for those non-imitative processes. They trained demonstrator pigeons to obtain food, either by pecking at a treadle (a flat metal plate located near the floor of the chanber), or by stepping on the treadle. Then they allowed observer pigeons to observe one of those behaviors (or the other). Finally, they allowed the observers to operate the treadle with either response. Zentall et al. found that the observers showed a significant tendency to operate the treadle in the same manner that they had observed it performed by the demonstrators. Using this procedure, an even stronger imitative response was found in Japanese quail, a species known to demonstrate imprinting (Akins and Zentall, 1996). The beauty of the two-action procedure is that it controls for social facilitation, stimulus enhancement, and learned affordances. That is, each group serves as a control for the other, the only difference being the manner in which the treadle was operated by the demonstrator, with its foot or with its beak.

Further research on imitation found that observers would not imitate if the demonstrator did not receive a reinforcer for their treadle response (Akins and Zentall, 1998). Nor would the observer imitate if, at the time of observation, it was not motivated by the reinforcer obtained by the demonstrator (i.e., if the observer had been prefed; Dorrance and Zentall, 2001).

Another interesting distinction related to imitation was suggested by Bandura (1969). In describing imitation by children, he distinguished between imitation and observational learning. Bandura claimed that imitation that occurred at the time of observation could be *reflexive* and was perhaps genetically predisposed (copying behavior sometimes referred to as response enhancement), whereas *observational learning* represented the internalization of the observed response, such that it could be performed at a later time.

Although in the research described above the observation and observer’s performance did not occur at the same time, not much time elapsed between the two. However, as part of a larger study (Dorrance and Zentall, 2001), observers that were tested 30 min following observation showed significant copying of the stepping or pecking behavior that they had earlier observed. Thus, according to Bandura, such copying should qualify as observational learning, a more cognitive behavior than “simple” imitation.

## Cognitive Biases

Certain human behaviors would be described as biased or even suboptimal because they appear to be inconsistent with basic principles of associative learning. Although these behaviors do not represent an accurate assessment of the contingencies of reinforcement, they are thought to result from the cognitive *misunderstanding* of the context. We have studied four of these in pigeons: Justification of effort (a version of cognitive dissonance), base rate neglect, unskilled gambling behavior, and sunk cost (the tendency to persist in a task based on past investment, rather than the future contingencies of reinforcement).

### Justification of Effort

When humans behave in ways that are inconsistent with their beliefs it is thought to create *cognitive dissonance*. This dissonance may be a social phenomenon resulting from an attempt to avoid being considered a hypocrite. Do animals have beliefs? If so, are they concerned about the consistency between their beliefs and their behavior? How would one go about evaluating their beliefs to determine whether they are consistent with their behavior? And how would one measure the presumed dissonance that might result from that inconsistency?

One version of cognitive dissonance, called *justification of effort*, may provide a tractable approach to study this behavior in animals. *Justification of effort* is the tendency to prefer reinforcers that one has worked harder to obtain. If the reinforcers are of equal value, a preference should not be found. In fact, one might expect that if one had to work hard for a reinforcer, it might reduce the value of the reinforcer and thus, it should not be preferred. If, however, there is a tendency to justify the effort put into obtaining the reinforcer, the reinforcer might be preferred. Cognitive dissonance theory would suggest that if one had to work harder to obtain the reinforcer, it must have more value. If not, the theory suggests, why did one work so hard to obtain it.

To study justification of effort in pigeons Clement et al. (2000) trained them to peck a white light. On half of the trials, the pigeon was required to peck the white light once, and then it changed to red. A single peck to the red light was reinforced. On the remaining trials, the pigeon was required to peck the white light 20 times, and then it changed to green. A single peck to the green light produced the same reinforcer. The purpose of the red and green lights was to have a way to distinguish between the two conditions of reinforcement because the reinforcers were exactly the same.

When Clement et al. (2000) tested the pigeons by giving them a choice between the red and green lights, the pigeons showed a significant preference for the green light, the color that they had to work harder to obtain (see also Friedrich and Zentall, 2004). Furthermore, they did so independently of the number of times they had to peck the white light on the test trial.

Other research indicated that other relatively less preferred events had the same effect. For example, pigeons generally prefer immediate over delayed reinforcement. However, they preferred a stimulus that followed a delay over one that did not (DiGian et al., 2004). Similarly, pigeons prefer food over the absence of food, yet they preferred a stimulus that followed the absence of food, over a stimulus that followed food (Friedrich et al., 2005).

Although this procedure fits the design of a justification of effort experiment, given that it was conducted with pigeons, one might not be inclined to interpret the results in terms of an inconsistency between the pigeon’s belief (that fewer pecks are better than more pecks) and its behavior (having pecked many more times to obtain the same reinforcer). Instead, one is likely to consider the preference in terms of a more behavioral mechanism. A likely alternative mechanism is positive contrast. In the case of 1 vs. 20 pecks, it would be the contrast between the effort expended in responding to the white stimulus and the appearance of the signal for reinforcement. One can think of the contrast in terms of frustration that occurs on the high effort trials, that is relieved upon the appearance of the stimulus signaling reinforcement.

If this effect occurs in pigeons, could positive contrast also account for examples of justification of effort in experiments with human subjects? If so, one might expect humans to show a similar effect when trained on the task we used with pigeons. Alessandri et al. (2008) tested this procedure with children using computer mouse clicks as the response requirement and found similar results, a preference for the stimulus that followed greater effort. Furthermore, Klein et al. (2005) used a similar procedure with human adults and found the same result. Interestingly, when the adult subjects were asked why they had chosen the stimulus that they had worked harder to obtain, most of them said that they did not know. This finding suggests that the bias to prefer the stimulus that the subjects had worked harder for was learned implicitly (unconsciously), and it suggests that it is likely to be a mechanism similar to that used by pigeons.

### Base-Rate Neglect

**FIGURE 9:**
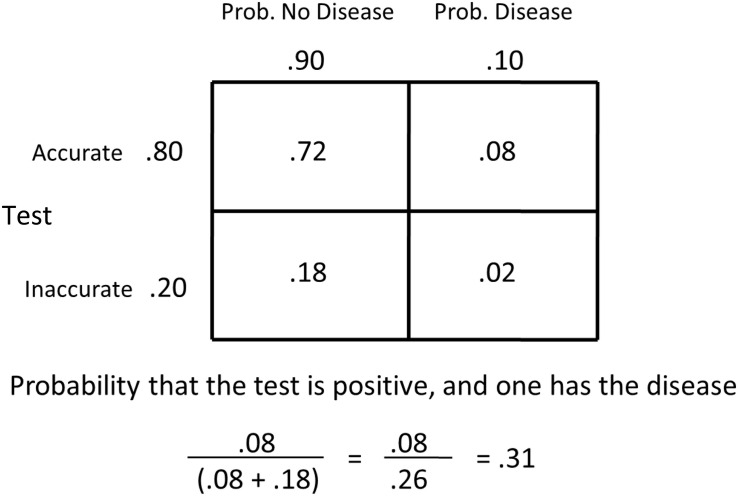
Matrix representing the probability of having and not having the disease by the accuracy of the test. The probability of having the disease equals the probability of having the disease, given the test is accurate, divided by the sum of the probabilities of having the disease, given the test is accurate, plus the probability of not having the disease, given that the test is inaccurate.

Base-rate neglect is a bias that fails to sufficiently include the original rate of a probable event. For example, let’s say that given certain symptoms, the probability of having the flu is 0.10. Let’s say, as well, that there is a test that is 80% accurate in diagnosing the flu. The test gives false positives 20% of the time and false negatives 20% of the time. Let’s say a patient tests positive, what is the likelihood that they have the flu? Many people would say 80% (or maybe somewhat less). In fact, the probability of having the flu is much less (see Figure 9). Given the probabilities described, it is the probability of an accurate diagnosis of the flu (0.08), divided by the sum of the accurate diagnosis of the flu (0.08) plus the probability of a false positive (an inaccurate diagnosis that it is the flu, 0.18). Thus, given a positive test, the probability that the patient actually has the flu is better than without the test (0.10), but it is actually only.31!

**FIGURE 10:**
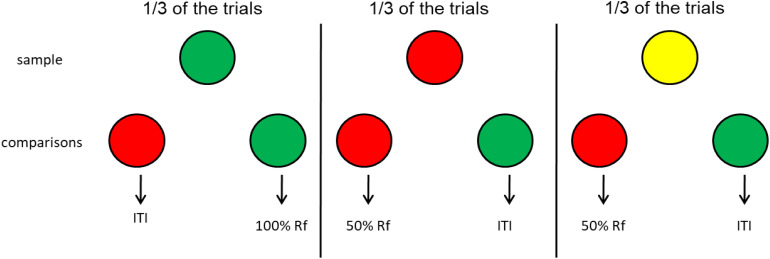
Pigeon base rate neglect experiment (Zentall et al., 2008). Pigeons had an equal probability of getting a red, green, or yellow sample. However, the green comparison was correct when the sample was green and correct choice was reinforced 100% of the time, and the red comparison was correct when the sample was red or yellow but correct choice was reinforced only 50% of the time. Thus, in the absence of memory for the sample, the probability of reinforcement associated with each of the comparisons (the base rate) was 33% for both comparison stimuli. Yet, with increasing delay between the offset of the sample and the onset of the comparison stimuli, the pigeons showed a strong preference for the red comparison stimulus.

How would one create a simulation of base-rate neglect for animals? The idea would be to create a condition in which, similar to the flu test, errors can occur. One such task might be delayed matching to sample involving, for example, red and green comparison stimuli equally associated with reinforcement (Zentall et al., 2008; see Figure 10). This would be considered the base rate because in the absence of the sample (the flu test) the red and green comparison stimuli should be equally associated with reinforcement. However, the samples (representing the flu test) would not be equally presented. On one third of the trials, the sample stimulus is green, and choice of the green comparison stimulus is reinforced. On another third of the trials, the sample stimulus is red, but correct choice of the red comparison stimulus is reinforced only 50% of the time. On the final third of the trials, the sample stimulus is yellow and correct choice of the red comparison stimulus is reinforced only 50% of the time. Thus on 1/3 of the trials choice of the green comparison is reinforced and on 2/3 of trials choice of the red comparison stimulus is reinforced but only 50% of the time, thus equally often as the green comparison.

Thus, when the sample stimulus (the flu test) is available, reinforcement can be obtained 67% of the time (all of the 33% of the trials with green samples and half of the 67% of the trials with red or yellow trials). But what should the pigeon do in the absence of memory for the sample? Which comparison should the pigeon choose? In the absence of a sample, because each of the comparison stimuli would be associated with 33% reinforcement, there should be no bias. This is the base rate. In training, however, the red comparison would have been chosen twice as often as the green comparison. Thus, in spite of the fact that correct choice of the red comparison was reinforced only 33% of the time, when there was a delay between the offset of the sample and onset of the comparison stimuli, the pigeons showed a strong preference for the red comparison stimulus. That is, they showed clear evidence of base-rate neglect. Similar findings were reported by Zentall and Clement (2002) and DiGian and Zentall (2007) using somewhat different designs. Thus, in these experiments, the pigeons were unduly influenced by the frequency with which they had responded to the two comparison stimuli (the equivalent of the accuracy of the flu test in the human example).

### Unskilled Gambling

When humans are engaged in unskilled gambling (e.g., slot machines, lotteries, roulette) their choice is almost always suboptimal (their investment is almost always greater than the return). Those who engage in such activities claim that they do it because gambling is entertaining. Thus, generally one would not expect non-human animals to engage in such behavior. If animals are hungry and working for food, entertainment should not be a factor. Furthermore, optimal foraging theory (Stephens and Krebs, 1986) proposes that animals have evolved to forage for food in the most effective way because more efficient foragers would survive and reproduce better.

**FIGURE 11:**
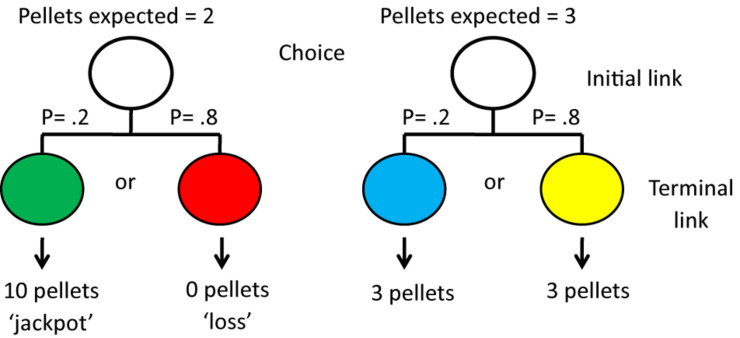
Pigeons were given a choice between the left side and the right side. If they chose the left side there was a 20% chance that they would receive a green light followed by 10 pellets of food but an 80% chance that they would receive a red light followed by no food. If they chose the right side, whether they received a blue light or a yellow light they always received 3 pellets of food. Thus, although they received 50% more food if they chose the right side, they showed a strong preference for the left side. After Zentall and Stagner (2011).

Research with pigeons, however, suggests otherwise. For example, Spetch et al. (1990) found that some pigeons preferred an alternative that provided a signal for 50% reinforcement (the gamble) over an alternative that provided a signal for 100% reinforcement (the non-gamble). In another experiment, involving manipulation of the magnitude of reinforcement, perhaps a better analog of human gambling behavior, Zentall and Stagner (2011) gave pigeons a choice between two alternatives. Choice of one alternative, 20% of the time, gave the pigeon a green light signaling that it would receive a “jackpot” of 10 pellets of food, but 80% of the time it would receive a red light signaling that it would get no food. Choice of the other alternative, 100% of the time, gave the pigeon a light signaling that it would get 3 pellets (the non-gambling option; see Figure 11). In this experiment, the pigeons showed a very strong preference for the gambling option that provided the pigeons with an average of 2 pellets per trial, over the non-gambling option that provided the pigeons with 3 pellets per trial (see also Stagner and Zentall, 2010).

These results suggest that the pigeons were not choosing between the values of the alternatives at the time of choice but between the value of the *signals for reinforcement* that followed that choice (Smith and Zentall, 2016). Thus, they appeared to be choosing, not between an average of 2 pellets vs. 3 pellets but between the occasional 10 pellets and 3 pellets. Interestingly, problem gamblers show a similar bias. When the value of a lottery is described in the media, the amount of a winning ticket is announced but rarely is one privy to the very low probability of such a win.

The theory that is the value of the signal for reinforcement that determines the value of the choice suggests that the signal for non-reinforcement, the one that occurred on 80% of the choices of the suboptimal alternative, has little inhibitory effect on choice. This hypothesis was confirmed by Laude et al. (2014), who found virtually no inhibition to the stimulus associated with the absence of reinforcement.

The hypothesis that it is the value of the signal for reinforcement that determine choice, was further tested by Case and Zentall (2018). They gave pigeons a choice between 50% signaled reinforcement and 100% signaled reinforcement. The hypothesis proposed by Smith and Zentall (2016) that it is the value of the signal for reinforcement that determines choice suggests that pigeons should be indifferent between the two alternatives, because although the choice of the 50% reinforcement alternative provided only half as much reinforcement as the 100% reinforcement alternative, both of the signals for reinforcement were associated with the same 100% reinforcement. Surprisingly, after a large amount of training on this task, the pigeons actually developed a significant preference for the suboptimal, 50% reinforcement, alternative!

Thus, it appears that there is a second mechanism involved in pigeons’ suboptimal choice. Case and Zentall (2018) proposed that positive contrast between the expected probability of reinforcement at the time of choice and the probability of reinforcement signaled by the conditioned stimulus (when it occurred) was responsible for the suboptimal preference.

To test the hypothesis that positive contrast is responsible for the preference for 50% reinforcement alternative over the 100% reinforcement alternative, Zentall et al. (2019) reduced the presumed amount of contrast, by *increasing* the probability of the signal for reinforcement associated with the suboptimal alternative from 50 to 75%. Thus, instead of a change in the probability of reinforcement from 50% at the time of choice to 100% upon the appearance of the conditioned stimulus (a difference of 50%) there was only a 25% difference. In spite of fact that the suboptimal alternative was now associated with *more* reinforcement (75%), there was a significant *reduction* in the preference for the suboptimal alternative. Thus, positive contrast between what is expected and what occurs appears to make an important contribution to the choice of the suboptimal alternative.

The positive contrast that pigeons show when the value of the conditioned reinforcer exceeds the expected value of choice may help to explain why humans engage in unskilled gambling. The few times that human gamblers win (or perhaps even imagine winning) may provide positive contrast (the feeling that gamblers express of being entertained) similar to that of pigeons.

### The Sunk Cost Fallacy

A sunk cost is an expenditure of resources that has already occurred. The sunk cost fallacy occurs when one allows a sunk cost to determine the future investment of resources. According to economic theory, the decision to invest further in a project should depend solely on the future likelihood of its success. However, humans often continue to invest in a losing project to avoid feeling that the project was a failure, but further investment is often likely to produce additional losses. The sunk cost fallacy also may result from the cultural admonition to avoid wasting resources. But in the case of a bad investment, the resources expended are already lost. Behavioral economists often point to the sunk cost fallacy as evidence that humans do not always behave rationally (Arkes and Blumer, 2000). Non-human animals, however, should be sensitive to future reinforcement contingencies and should not be affected by cultural factors like the sunk cost fallacy.

However, several experiments have demonstrated the sunk cost fallacy in animals. For example, Navarro and Fantino (2005) examined the sunk cost effect in pigeons in which, on each trial there was a 50% chance that a small number (10) of pecks would be required for reinforcement and a decreasing probability that many more responses (40, 80, or 160) would be required. At any time, the pigeon could peck a different response key that would start a new trial, thereby potentially getting a trial with a smaller number of pecks to reinforcement. The optimal strategy would be to peck 10 times and, in the absence of reinforcement, start a new trial. Surprisingly, the pigeons in that study generally persisted and rarely choose to start a new trial (see also Magalhāes and White, 2014).

This task is similar to the economic sunk cost effect with humans because in both cases there is some uncertainty about the likelihood that persistence will not pay off. For the pigeon, after 10 pecks, it would be best to start a new trial, however, it is possible that persisting will produce food after 40 more pecks, whereas by starting a new trial it could take 80 or even 160 pecks to produce food. Also, starting a new trial required the pigeon to stop pecking, move to the other response key, peck it, and then move back to the original response key.

Pattison et al. (2012) asked if pigeons would show a sunk cost effect even if there was no uncertainty about the results of persisting and no differential cost to switch to the other response key. In one experiment, they first trained pigeons to peck a green key 30 times for food on some trials and peck a red key 10 times for food on other trials. Then they trained the pigeons to peck a green light on a side key a variable number of times to turn off the green light and light a white key in the middle. A single peck to the white key relit the green key and also lit a red key on the other side of the white key. Now from the middle white key, the pigeon could choose to go back and peck the green key enough times to total 30 pecks (the initial investment plus the remaining pecks had to equal 30) to obtain a reinforcer. Or it could switch to the red key for 10 pecks to obtain a reinforcer. The question was, would the pigeon switch to the red key that required 10 pecks for reinforcement when going back to the green key meant it would have to make more than 10 pecks for reinforcement.

The results indicated that when the pigeons had invested as few as 10 pecks to the green key, they preferred to return to the green key for the remaining 20 pecks, rather than switch to the red key for 10 pecks. Under these conditions, at the time of choice, there should have been no uncertainty about the number of remaining pecks and the pigeons were equally distant from the green and red keys. Thus, they were biased to return to the green key, even though it required more pecks. Only when there were no initial pecks to the green key (no prior investment) did the pigeons prefer the red key over the green key. Thus, much like humans, the pigeons preferred to complete a task already started, rather than switch to another task.

The sunk cost fallacy may be related to a human gambling phenomenon, known as *chasing losses*. When gamblers start to lose, they often show a tendency to keep gambling, with the intent to recoup the money that they have lost. As a result, they typically get further into debt.

### Uniquely Human Fallacies

There are several fallacies shown by humans that other animals do not appear to show, fallacies that appear to result from human experience or knowledge, or that may be cultural in nature. One of these is the Monte Carlo fallacy. Another is the near miss fallacy.

#### The Monte Carlo Fallacy

The *Monte Carlo* or *gambler’s fallacy* occurs when, over the short-term, a series of events appears to show a bias for one outcome over the other. For example, if one flips a coin 4 times and each time it comes up heads, many people believe that the probability that it will come up heads again is now less than 50%. They believe that it should come up tails, to make up for the unlikely outcome of 4 heads in succession. But the coin tosses are independent—the coin has no memory of its past behavior. So, this is a fallacy.

Most animals live in an often-changing world but one in which purely random, independent events are rare. Thus, their sensitivity to short-term changes in the probability of events is likely to bias them in the direction of those changes, rather than in the reverse direction. That is, if they are exposed to random events in which a particular event occurred 4 times in succession (e.g., reinforcement occurring for a response to the left key of a response panel), they are likely to show a greater tendency to make that same response again, rather than make a response to the other alternative. Pigeons are not likely to *know* that the probability of a coin toss is equally likely to be heads as tails.

#### The Near Miss Fallacy

The near miss fallacy (more accurately called the near hit fallacy) can most easily be seen in the way that slot machines function. Traditional slot machines have three spinning reels. The player wins if the images on the three wheels match when the reels stop spinning. All other patterns of images on the three reels usually indicate a loss. The interesting case is when the first two images match but the third one does not. This is referred to as the near hit outcome. If the first two reels do not match, it is already a loss, but if the first two reels match, a win is still possible. For this reason, it has been found that, with equal probability of winning, people prefer to play slot machines with a higher rate of near hit outcomes (MacLin et al., 2007).

**FIGURE 12:**
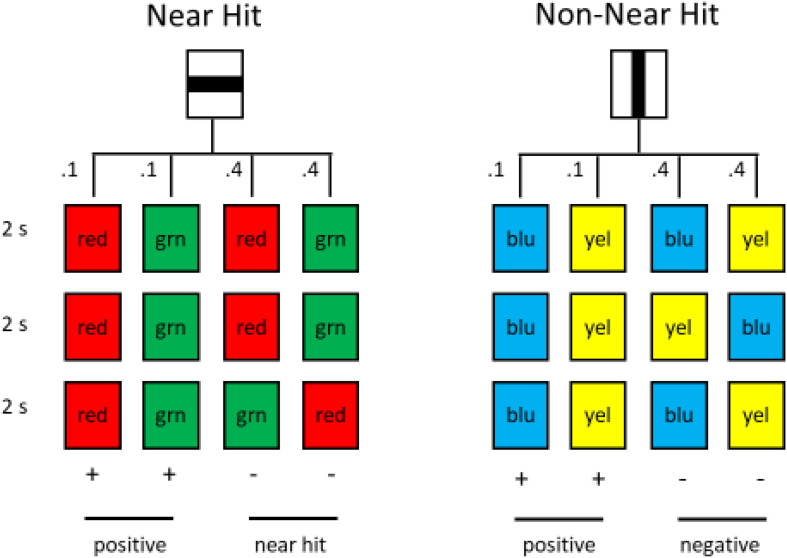
Pigeons had a choice between the horizontal line and the vertical line. Choice of the horizontal line led to (1) the successive presentation of three red or three green stimuli, each with a probability of 0.10 or (2) the successive presentation of two red and one green or two green and one red stimulus, each with a probability of 0.40. Choice of the vertical line led to (1) the successive presentation of three blue or three yellow stimuli, each with a probability of 0.10 or (2) the successive presentation of blue, yellow, blue or yellow, blue, yellow, each with a probability of 0.40 (after Fortes et al., 2017).

The slot-machine task easily can be modified for use with pigeons. In several experiments, pigeons were trained on such a task, with two alternatives, equated for wins and losses (Stagner et al., 2015; Fortes et al., 2017). The pigeons could choose between an alternative with near hit trials and another with a different pattern of losses (see Figure 12). Unlike humans, however, the pigeons tended to avoid the alternative with the near hit trials. Apparently, for pigeons the similarity of the appearance of near hit losses to win trials actually devalued the effects of a win. That is, to a pigeon it may be that red—red—green appears more similar to red—red—red than to, for example, red—green—red.

It is interesting to speculate about the mechanisms responsible for the difference between humans and pigeons with this task. One possibility is that humans have considerable experience with tasks in which losses, similar to a near hit, represent progress toward a goal.

Consider trying to get a basketball into a hoop. Initially, one might miss the hoop entirely. With practice one should be able to get closer to putting the ball into the hoop, but still not get it in. That improvement, still involving losses, would be evidence that one is making progress. Thus, the near hit in basketball represents an improvement in one’s performance. In the slot machine task in which there is no skill, however, the near hit does not represent progress. To a gambler, however, it may feel like improvement. It would be interesting to know if pigeons that were trained on several tasks in which gradual learning of skill was needed, would also develop a preference for near hit trials when transferred to a slot-machine like task.

## Conclusion

The research described in the present article, together with a great deal of related research on comparative cognition, suggests that Macphail’s hypothesis that all vertebrates have similar cognitive capacities may not be as implausible as it may at first appear. Once one has accounted for important differences in contextual variables concerned with perception, motor skills, and motivation, many of the presumed differences may be more quantitative than qualitative. By its nature, it may not be possible to demonstrate that Macphail’s hypothesis is false because one may not ever be able to ensure that the contextual variables are all appropriate for the species in question. However, whatever the outcome of the quest to test Macphail’s challenge, I have found it to have resulted in a wealth of informative research on comparative cognition.

## Author Contributions

TZ prepared the manuscript.

## Conflict of Interest

The author declares that the research was conducted in the absence of any commercial or financial relationships that could be construed as a potential conflict of interest.
